# Growth Dynamics of Australia's Polar Dinosaurs

**DOI:** 10.1371/journal.pone.0023339

**Published:** 2011-08-03

**Authors:** Holly N. Woodward, Thomas H. Rich, Anusuya Chinsamy, Patricia Vickers-Rich

**Affiliations:** 1 Museum of the Rockies, Montana State University, Bozeman, Montana, United States of America; 2 Museum Victoria, Melbourne, Victoria, Australia; 3 Zoology Department, University of Cape Town, Rhodes Gift, South Africa; 4 School of Geosciences, Monash University, Clayton, Victoria, Australia; Raymond M. Alf Museum of Paleontology, United States of America

## Abstract

Analysis of bone microstructure in ornithopod and theropod dinosaurs from Victoria, Australia, documents ontogenetic changes, providing insight into the dinosaurs' successful habitation of Cretaceous Antarctic environments. Woven-fibered bone tissue in the smallest specimens indicates rapid growth rates during early ontogeny. Later ontogeny is marked by parallel-fibered tissue, suggesting reduced growth rates approaching skeletal maturity. Bone microstructure similarities between the ornithopods and theropods, including the presence of LAGs in each group, suggest there is no osteohistologic evidence supporting the hypothesis that polar theropods hibernated seasonally. Results instead suggest high-latitude dinosaurs had growth trajectories similar to their lower-latitude relatives and thus, rapid early ontogenetic growth and the cyclical suspensions of growth inherent in the theropod and ornithopod lineages enabled them to successfully exploit polar regions.

## Introduction

During the Early Cretaceous, the state of Victoria, Australia, lay within the Antarctic Circle between the paleolatitudes of 75°S and 80°S [1], [2]. Although there is no firm consensus regarding the severity of environmental conditions [3], [4], this region certainly experienced prolonged periods of light and dark [1] with mean annual air temperatures ranging between −6°C and +10°C [5]–[7]. Because of this, it has been hypothesized that dinosaurs inhabiting this unique environment were physiologically distinct from their counterparts at lower latitudes [8], [9] in order to survive the environmental extremes. To date, no fully articulated dinosaur specimens have been recovered from Victoria. Often, specimens are quite fragmentary, making taxonomic assignment impossible. Interestingly, the majority of identifiable Early Cretaceous (Aptian-Albian) dinosaur material collected from Victoria is from small-bodied ornithopods (i.e., “hypsilophodontids”). As “hypsilophodontids” are considered a paraphyletic grouping [10] the use of the term here is informal and refers to small bodied ornithischians possessing basal ornithopod characteristics. Dinosaurs assigned to this paraphyletic group have been formally described from every continent except Antarctica.

Because fossil bone histology provides insights into the growth dynamics of extinct animals [9], histological analysis can be reliably used to assess hypotheses related to physiology. A previous osteohistologic study of femora from a Victorian polar theropod and hypsilophodontid suggested that these dinosaurs were behaviorally different. Presence of zonal bone was interpreted to mean that the theropod likely hibernated during the severe winters, whereas the absence of zonal bone in the hypsilophodontid suggested that it remained active and continued to grow throughout the winter [8]. This hypothesis continues to influence interpretations of polar dinosaur behavior and physiology (e.g., [11], [12]–[15]).

Since that initial study, the number of hypsilophodontid fossils collected from Victoria has increased substantially, thus permitting a larger sample for osteohistologic examination. The current assessment includes nine femora and eight tibiae from hypsilophodontids as well as one theropod femur. Although the number of Early Cretaceous hypsilophodontid genera may be as high as six [1], [3], it has recently been argued that the diversity was much less [16], but this remains unresolved. Regardless, as only limb elements are used in this study the specimens are not identified beyond “hypsilophodontid”. The theropod femur is tentatively assigned to *Timimus* based on similarities to the type femur [17], as well as to the femur examined by Chinsamy [8]. *Timimus* may either be an ornithomimid [17] or a dromaeosaurid [16].

Now that more dinosaur fossils from Victoria are available, the goal of this research is to provide a more robust analysis of polar dinosaur osteohistology and to test the hypothesis that there is microstructural evidence suggesting at least some polar dinosaurs hibernated. Results of this study allow evaluation of ontogenetic changes in bone tissue, enable comparisons with the bone microstructure of lower-latitude hypsilophodontid and theropod relatives, and lead to a new hypothesis to explain the successful radiation of dinosaurs in high-latitude environments.

## Results

**Figure 1:**
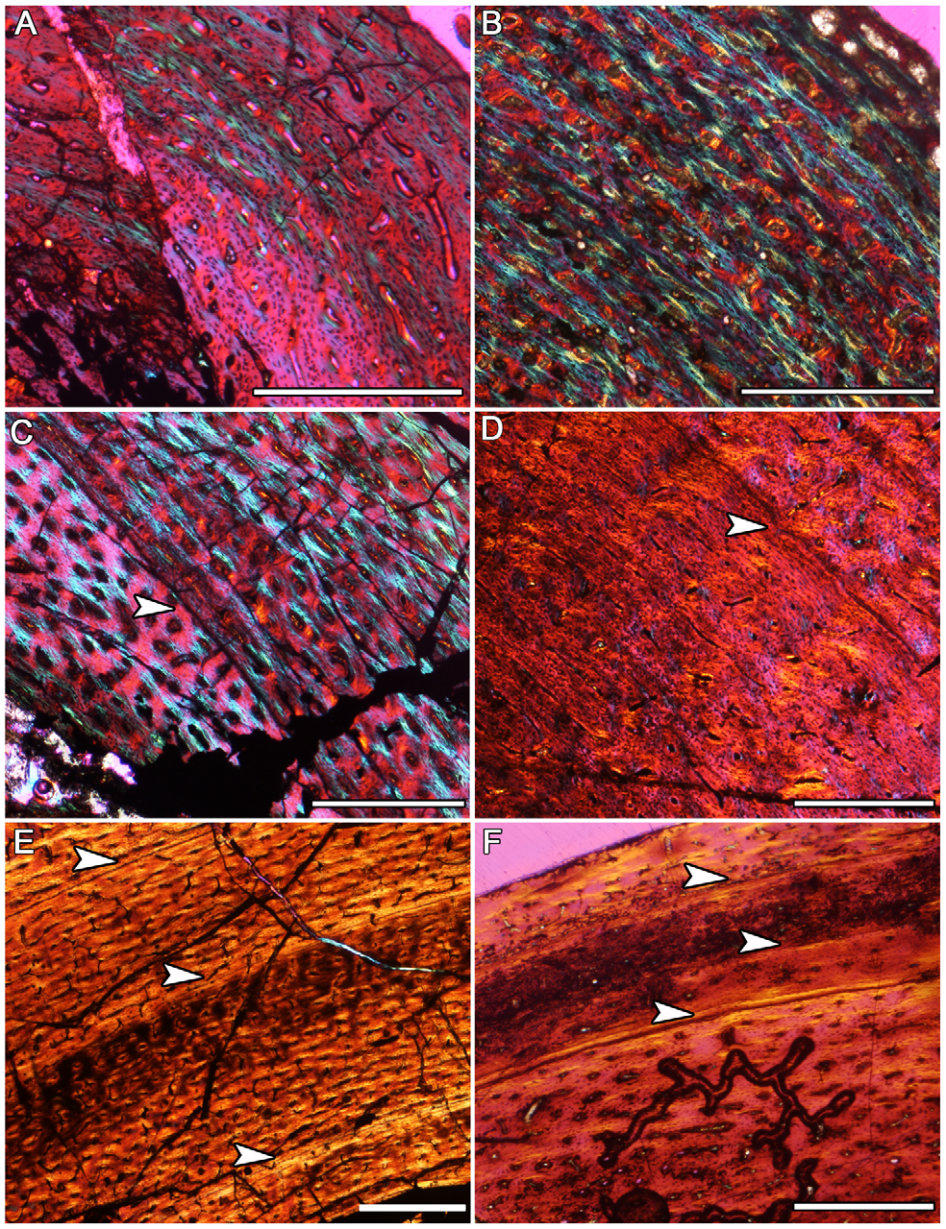
Hypsilophodontid ontogenetic bone microstructure. The bone microstructures observed in hypsilophodontids from Victoria (left column) suggest ontogenetic status, and strongly resemble microstructures observed in hypsilophodontids from lower latitudes (right column; Museum of the Rockies- MOR; Brigham Young University- BYU). Arrows indicate LAGs. Scale bars, 500 µm. A) Femur (NMV 216768) diaphyseal section from a skeletally immature polar hypsilophodontid. Growth marks are absent and tissue consists of rapidly deposited, disorganized woven fibers. This resembles the femoral diaphyseal section in B), from an *Orodromeus* (MOR 407). C) Diaphyseal section of a femur (NMV 208495) showing one LAG within fast growing woven tissue. LAGs within woven tissue are also observed in D), a femur from a *Dryosaurus* (BYU 13312). E) Diaphyseal section of a tibia (NMV 228434) consisting of three well-defined LAGs within loosely parallel-fibered cortex, representing a slowly growing individual approaching skeletal maturity. A similar microstructural pattern is observed in F), an *Orodromeus* tibia (MOR 973) possessing LAGs within a parallel-fibered matrix.

**Figure 2:**
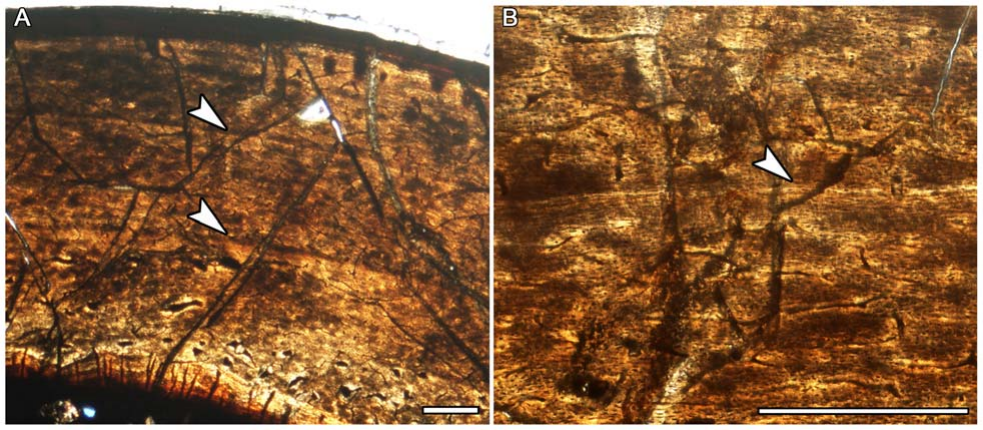
Bone microstructure of polar hypsilophodontid approaching skeletal maturity. Arrows indicate annuli. Scale bars, 500 µm. A) Diaphyseal section of femur NMV 177935 consisting of parallel-fibered tissue with two growth marks within the cortex. B) Detail of inner growth mark showing a distinctive annulus terminating with a bright line.

Almost every hypsilophodontid long bone examined had cyclical growth marks in the form of lines of arrested growth (LAGs), or annuli followed by “bright lines” (which are probably equivalent to LAGs) (Table 1). Growth marks were fully traceable around the circumference of the thin sections except where truncated by secondary osteons, crushing, or poor preservation. The smallest hypsilophodontid femur and tibia studied displayed well-vascularized, woven bone tissue (Figure 1A) typical of rapidly growing animals [18] and lacked growth marks. The largest hypsilophodontid specimens possessed parallel-fibered tissue in the outer cortex (Figure 1E; Figure 2) and at least seven LAGs are visible (Table 1), although more may have been lost to remodeling. Included among the samples containing growth marks is the same hypsilophodontid femur (NMV 177935) examined by Chinsamy et al. [8]. Contrary to the original three sections (which were reexamined, and no LAGs were visible), the two new sections made for this report revealed interruptions in growth in the form of annuli followed by bright lines (Figure 2). As with LAGs, bright lines suggest hypermineralized tissue layers representing a brief hiatus in growth [18].

**Table 1:** Data for Australian polar dinosaur fossils included in this study.

Museum Number	Element	Element Length (cm)	Growth Marks Preserved	Locality Name	Formation	Locality Coordinates
NMV P186326	Femur	18.5	3 annuli	Dinosaur Cove East	Eumerella	38°46′53±1″S, 143°24′14±1″E
NMV P177935	Femur	20.8*	2 annuli	Dinosaur Cove East	Eumerella	38°46′53±1″S, 143°24′14±1″E
NMV P150054	Femur	15.5*	3 annuli	Eagle's Nest	Wonthaggi	38°40.5′S, 145°40.25′E
NMV P216768	Femur	4.7	0	Flat Rocks	Wonthaggi	38°39′40±2″S, 145°40′52±3″E
NMV P208495	Femur	13*	1 LAG	Flat Rocks	Wonthaggi	38°39′40±2″S, 145°40′52±3″E
NMV P199058	Femur	14.5*	3 LAGs	Flat Rocks	Wonthaggi	38°39′40±2″S, 145°40′52±3″E
NMV P221151	Femur	16*	5 LAGs	Flat Rocks	Wonthaggi	38°39′40±2″S, 145°40′52±3″E
NMV P180892	Femur	31.5*	7 LAGs	Rotten Point	Eumerella	38°46′55±1″S, 143°24′3±1″E
NMV P186047	Femur	12.8	4 annuli	Slippery Rock	Eumerella	38°46′54±1″S, 143°24′15±1″E
NMV P186334	Tibia	17.2	4 annuli	Dinosaur Cove East	Eumerella	38°46′53±1″S, 143°24′14±1″E
NMV P210062	Tibia	11.5	0	Flat Rocks	Wonthaggi	38°39′40±2″S, 145°40′52±3″E
NMV P208204	Tibia	14.4*	2 LAGs	Flat Rocks	Wonthaggi	38°39′40±2″S, 145°40′52±3″E
NMV P208336	Tibia	19.4*	4 LAGs	Flat Rocks	Wonthaggi	38°39′40±2″S, 145°40′52±3″E
NMV P199133	Tibia	16	3 LAGs	Flat Rocks	Wonthaggi	38°39′40±2″S, 145°40′52±3″E
NMV P208189	Tibia	18.8	2 LAGs	Flat Rocks	Wonthaggi	38°39′40±2″S, 145°40′52±3″E
NMV P228434	Tibia	21.8	7 LAGs	Flat Rocks	Wonthaggi	38°39′40±2″S, 145°40′52±3″E
NMV P228360	Tibia	20	6 LAGs	Point Lewis	Eumerella	38°50′9 to 12″S, 143°34′53 to 57″E

Estimates of length indicated by asterisk. NMV: Museum Victoria, Melbourne, Victoria, Australia.

**Figure 3:**
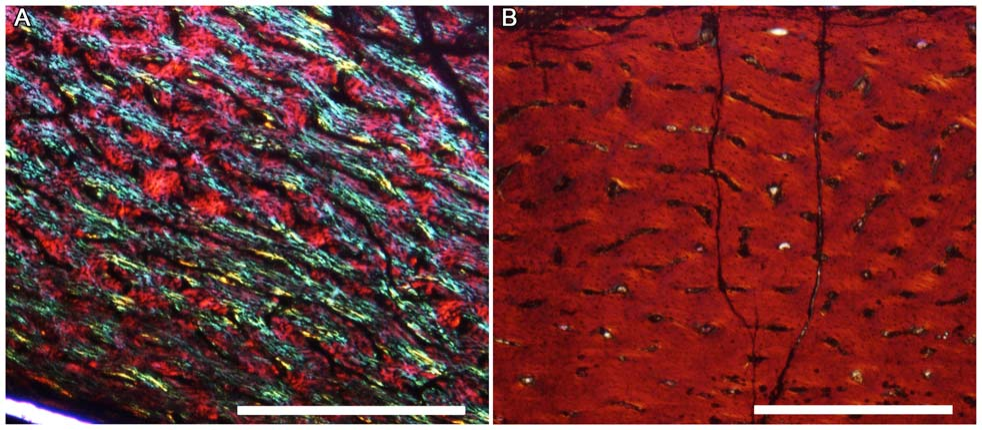
Theropod osteohistology. Diaphyseal sections of high-latitude and lower-latitude theropods. Scale bars, 500 µm. A) Theropod femur diaphyseal section from Victoria (NMV 186323). Note the well-vascularized cortex consisting of rapidly deposited tissue. B) *Troodon* tibia (MOR 563) from Montana, USA, showing a similarly well-vascularized cortex and rapidly deposited tissue.

The theropod femur (NMV 186317) sectioned by Chinsamy et al. [8] was estimated at 44 cm in length and considered skeletally mature due to its microstructure. In contrast, the theropod femur (NMV 186323; Figure 3A) examined in this study is considerably smaller (19.3 cm in length; Table 1), and as in the young hypsilophodontids, the bone microstructure is well-vascularized with primary osteons in woven tissue- typical of rapidly growing juvenile vertebrates [18]. There were no LAGs within the cortex of this specimen.

## Discussion

Studies performed on extant taxa imply that growth mark periodicity is annual (e.g., [19], [20]–[22]), although the degree to which growth decreases is determined by the type of mark. Lines of arrested growth indicate periodic suspensions in bone mineral deposition and thus suspensions in long bone growth; annuli indicate periodic decreases in the rate at which mineral is deposited, hence only a periodic decrease in overall growth rate [23]. Among the hypsilophodontids, the occurrence of either LAGs (suspension of growth) or annuli followed by bright lines (dramatically slowed growth before suspension) in different specimens suggests some measure of inter-specific, intra-specific, or inter-elemental variation in the degree to which growth cyclically decreased.

As the smallest hypsilophodontid femur and tibia consist of woven-fibered tissue and lack growth marks (Figure 1A; Table 1), these bones may be from individuals less than one year of age. In larger individuals, up to three growth marks interrupt the deposition of woven-fibered tissue (Figure 1C), indicating an initial 3 year period of rapid growth [23]. Thereafter, parallel-fibered tissue, reduced vascularity, and multiple LAGs (Figure 1E) reveal that growth slowed significantly but continued for several years until skeletal maturity was reached. Sexual maturity may be marked by the transition from rapid to more slowly formed tissue [24] at three years of age. The tissues and growth patterns observed in the polar hypsilophodontids are also noted in the bone microstructure of low-latitude basal ornithopods such as *Orodromeus*, *Dryosaurus*, and *Scutellosaurus* (Figure 1B, D, F; [10], [25]).

The large theropod femur (NMV 186317) examined by Chinsamy et al. [8] consisted of a vascularized woven-fibered inner-cortex that changed to poorly vascularized, parallel-fibered tissue after two LAGs. In all, nine LAGs were visible. This was interpreted as rapid growth for at least two years, and then likely the attainment of sexual maturity [24], after which growth slowed.

In contrast, the smaller theropod femur examined in this study (NMV 186323) had well vascularized, woven-fibered tissue throughout the cortex and lacked LAGs (Figure 3A). Although it is possible that LAGs were obliterated due to medullary expansion, the small size of the femur along with the bone tissue organization suggests that this dinosaur was growing quickly and that it was less than one year of age. The bone tissues observed in NMV 186317 and NMV 186323 strongly resemble the patterns of microstructural changes described in an ontogenetic study of the lower-latitude dromaeosaurid *Troodon formosus* (Figure 3B; [26]).

Despite the variability between LAGs and annuli within the hypsilophodontid samples, the microstructures and cyclical growth exhibited by both ornithopods and theropods from the high paleolatitude of southeastern Australia resemble patterns observed in dinosaurs from lower paleolatitudes, indicating similarities in growth dynamics and physiology. Although LAGs can form as a result of the slowed metabolic processes experienced during hibernation, they are not microstructural features exclusive to hibernators [23]. Therefore, contrary to the earlier hypothesis of Chinsamy et al. [8], we suggest that the presence of growth marks alone cannot be used to support a hibernating behavior.

While it is entirely likely that polar dinosaurs had specialized behavioral and morphological adaptations to cope with their environment, only a few possibilities can be tested with fossil bone microanalyses. For instance, several microstructure studies have demonstrated that Triassic and Jurassic archosaurs (including dinosaurs) exhibited cyclical suspensions in growth as well as high growth rates early in life [25], [27]–[31]. These characteristics, also observed in more derived archosaurs, are likely plesiomorphic for the archosauriform clade [23], [31] and would have allowed dinosaurs to establish themselves in ecological extremes without requiring a change in physiology: early rapid growth reduced the time dinosaurs were highly sensitive to predation and environmental stresses, while periodic suspensions in bone deposition conserved resources during seasonal stresses. Therefore, on the basis of the bone tissues evident in the polar hypsilophodontids and theropods, we propose that the high initial growth rates observed in these taxa, along with cyclical suspensions of growth (possibly coinciding with the dark winter months), are exaptations that could explain their successful exploitation of a hostile high-latitude ecosystem.

## Materials and Methods

### Ethics Statement

This research was sponsored by the 2010 East Asia and Pacific Summer Institutes program, jointly organized by the National Science Foundation (proposal number OISE-1015130) and the Australian Academy of Sciences. Access to dinosaur fossil collections at Museum Victoria (Melbourne, Victoria, Australia) was provided via a letter of invitation from Dr. Thomas Rich (Senior Curator of Vertebrate Paleontology and Paleobotany) and Museum Victoria permit number VP 2010/07.

### Procedure

Nine femora and eight tibiae from small bodied hypsilophodontid ornithopod dinosaurs and one femur from a theropod dinosaur were selected for histological analysis from the collections of Museum Victoria, Melbourne, Victoria, Australia (NMV). Isolated limb elements were collected from either the Wonthaggi Formation (Early Aptian) of the Strzelecki Group or the Eumerella Formation (latest Aptian-Early Albian) of the Otway Group (Table 1) [3], [4], [32]. The small theropod femur examined here (NMV 186323) is tentatively assigned to *Timimus hermani*, based on morphological similarities to the theropod femur (NMV 186317) sampled by Chinsamy et al. [8] as well as the type specimen (NMV P186303) [17]. However, the ornithopods are not identified beyond “hypsilophodontid”. The hypsilophodontid sample set includes the femur (NMV 177935) originally sampled by Chinsamy et al. [8], from which another section distal to the fourth trochanter was made. In addition, the three original thin sections made from NMV 177935 by Chinsamy et al. [8] were provided for comparison, and we verified that no growth marks were visible in these sections.

Samples containing the mid-diaphysis were removed from the shafts when possible and first processed using a procedure modified from Schweitzer et al. [33]. Each sample was next embedded with SpeciFix-40 (Struers) two-part epoxy resin and then prepared following Lamm [34]. Some thin section slices were mounted to frosted glass slides using two-part epoxy glue, while others were mounted to frosted plastic slides using cyanoacrylate glue. Within days of mounting the specimens onto glass slides the mounted surface developed a “cloudy” appearance suggesting poor adherence, while the plastic slides remained clear. Therefore, the exposed surfaces of the specimens mounted to glass slides were polished to a 600 grit finish and mounted to plastic slides with cyanoacrylate glue, “sandwiching” each specimen between a glass and plastic slide. The glass slide was then loaded onto glass slide chucks (Buehler Ltd.) and removed from the specimen using a 5-inch diameter diamond blade (Norton®) and an IsoMet 1000® Precision Saw (Buehler Ltd.). Some slides had already been polished to completion before the cloudiness occurred; these were soaked in xylene for clearing and coated with Polymount® (Polysciences Inc.) liquid cover slip medium, which penetrated underneath the specimen and restored the original visibility. Due to diagenetic staining of the fossils, many thin sections remained nearly opaque, even when polished very thin. In those cases, a mixture of IsoCut® (Buehler Ltd.) fluid and mineral oil was applied to the finished surfaces, greatly enhancing microstructure visibility. Completed thin section slides were photographed with either 4× or 10× objectives using a Nikon Optiphot-Pol polarizing microscope. Photomicrographs were taken with a Nikon DS-Fi1 digital sight camera, and thin section images were compiled using the NIS-Elements BR 3.0 software.
